# Helium Ion Microscopy and Sectioning of Spider Silk

**DOI:** 10.1155/2023/2936788

**Published:** 2023-05-22

**Authors:** Irina Iachina, Jonathan R. Brewer, Horst-Günter Rubahn, Jacek Fiutowski

**Affiliations:** ^1^NanoSYD, Mads Clausen Institute, University of Southern Denmark, Denmark; ^2^Department of Biochemisty and Molecular Biology, University of Southern Denmark, Denmark

## Abstract

Focused ion beams have recently emerged as a powerful tool for ultrastructural imaging of biological samples. In this article, we show that helium ion microscopy (HIM), in combination with ion milling, can be used to visualize the inner structure of both major and minor ampullate silk fibers of the orb-web weaving spider *Nephila madagascariensis*. The internal nanofibrils were imaged in pristine silk fibers, with little or no damage to the sample structure observed. Furthermore, a method to cut/rupture the fibers using He^+^ ions combined with internal sample tension is presented. This showed that the stretching and rupturing of spider silk is a highly dynamic process with considerable material reorganization.

## 1. Introduction

Bioimaging has become a vital tool with multiple applications within life science and other fields. Due to the diverse nature of biological samples, there are a large number of different bioimaging technologies, such as electron [1], ion [2–4], optical [5–8] or X-ray microscopy [9], magnetic resonance imaging (MRI) [10], ultrasonics [11], or techniques such as spectroscopy [12], and atomic force microscopy (AFM) [13]. Each of these techniques has characteristic strengths and weaknesses. Helium ion microscopy (HIM) has recently joined the class of charged particle microscopes, which includes all instruments that utilize a charged particle beam for precision imaging or patterning of a sample. The key to the system's performance is its source, with a single atom selected for imaging with the help of a beam-limiting aperture. Thanks to the small aperture diameters and a very low beam convergence angle, the helium ion microscope exhibits remarkably high lateral resolution in imaging (below 1 nm [14]) and an extremely large depth of field [15], five times greater than in a scanning electron microscope. Helium ions are more particle-like than electrons due to higher mass, so the spot size of the scanned beam is not limited by any diffraction aberrations [16].

In addition to the high-resolution HIM imaging, increasing the He^+^ ion dose or switching to the heavier Ne^+^ ions can modify the sample by “milling” off material from the area of interest [17, 18]. Traditional FIB-SEM with Ga+, compared to HIM, suffers from an order of magnitude lower milling resolution due to the larger sample beam interaction volume, degraded SEM imaging capabilities, and sensitivities to charging artifacts.

Due to spider silk's unique mechanical properties, considerable work has been done to understand the microscopic and nanoscopic structure of the silk. This has been done using techniques such as atomic force microscopy [19–23], light microscopy [24], scanning electron microscopy in combination with focused ion beam milling [22–25], and transmission electron microscopy [25, 26]. All those techniques have serious drawbacks, such as low resolution, complicated sample preparation, or being only surface sensitive, which limit their use for understanding the structural nature of spider silk. HIM has been shown to be an emerging technique for imaging of biological samples, making it an interesting tool for investigating spider silk [27–31].

In the present work, a combination of He^+^ ion milling and HIM is used to visualize the nanoscale structure of major (MAS) and minor (MiS) ampullate silk fibers from pristine samples of the orb-web spider *Nephila madagascariensis*. MAS is mainly used as the spiders' lifeline and for the radii spokes of the web, whereas MiS is used primarily for temporary scaffolding during web construction [32]. MAS and MiS fibers share gland morphology and some protein motifs but display different mechanical properties [33–35].

In the following, a novel method using He^+^ ion milling, together with sample tension, is used to cut/rupture the sample, giving access to an image of the internal structure of the spider silk.

## 2. Materials and Methods

MAS and MiS fibers were imaged using a Zeiss ORION NanoFab Helium Ion Microscope with SE detection (Zeiss, Oberkochen, Germany). Before HIM imaging, no surface modification or conductive coating was applied to the samples to preserve the surface information.

He^+^ imaging was performed at 25 keV beam energy, with a probe current ranging from 0.2 to 1 pA, and a scan dwell time of 1-2 *μ*s. Charge compensation was applied using a low-energy electron beam, a flood gun with 677 eV, and a flood time spanning from 10 *μ*m to 216 *μ*m. Sample working distance is 8.7 mm.

He^+^ surface sputtering was performed at 25 keV beam energy, with a probe current ranging from 1 to 7 pA, with a flood gun continuously on. The milling was controlled by NPVE software, with dwell time starting at 1 nC/*μ*m^2^ and a “standard” milling pattern with 50% to 20% overlap. Ne^+^ milling was performed with similar parameters for probe beam currents from 0.6 to 1 pA.

### 2.1. Silk extraction

Through forceful silk extraction, spider silk fibers were drawn from female Nephila madagascariensis spiders. After immobilising the spider, MAS or MiS fibers were, using tweezers, fastened to a reeling machine using double-sided tape. The spider silk was reeled at a constant speed of 7.7 mm*/*s^-l^.

### 2.2. Sample preparation

Using a tweezers and scissors, the extracted silk was carefully cut into a length to fit on top of aluminium specimen mounts (Plano GmbH, Germany) and mounted using carbon tape (Ted Pella, Inc., USA).

## 3. Results and Discussion

Major and minor ampullate silk (MAS and MiS) fibers from the orb-web weaving spider *Nephila madagascariensis* were fixed on carbon tape and raster-scanned with a He^+^ ion beam. Initial images of the silk showed no structure or contrast on the silk surface. This is most likely due to the outermost lipid layer, which coats the silk, causing surface charging of the sample.

**Figure 1:**
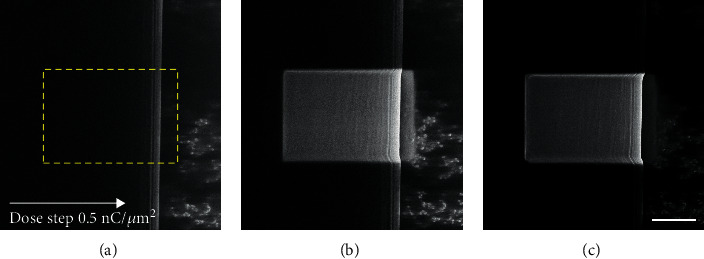
HIM images of a MAS fiber show the effect of increasing milling overtime in the marked area with an ion dose of 0.5nC/*μ*m^2^ between each successive image. It can be seen that the outer layers are etched away by the sputtering of He^+^ ions, and the inner structure of fibrils becomes visible. The scale bar is 1 *μ*m.

However, by continuously exposing a marked area of the sample to He^+^ ions, the outermost layers of the silk could be removed by surface sputtering.  Figure 1 shows layer-by-layer material removal of a MAS fiber where the inner nanofibrils in the silk become clearer as the outermost layer of the silk is removed, suggesting that the outer layers of the fiber are less conductive than the inner protein core. The images presented in Figure 1 are representative of multiple measurements of different spider silk fibers and types. Milling/cutting of biological samples with He^+^ ions and Ne^+^ ions of biological samples in connection with He^+^ ions has been demonstrated previously [18, 30]. For example, the microencapsulation of bacteriophages with a membrane emulsification process has been studied. Where the internal structure of the microcapsules was visualized with a combination of Ne-ion cross-sectioning and HIM imaging [36]. In the work we present here, we use currents five times lower than those used by Said et al. [28] which we believe considerably reduces possible ion and heat damage to the sample structures [37]. The sample was cut by sputtering with Ne^+^ ions to image the silk's inner structure. The ions could cut the sample; however, no clear inner structure could be seen, see Supplementary Figure s1. This was most likely due to the heavy Ne^+^ ions destroying the fine structures in the silk.

In Figure 1, a clear periodic structure of the fibrils is seen. They run parallel to each other and along with the silk fiber. The bright lines seen in the image are believed to be made by the edges of the fibrils, caused by the increased release of electrons from the edge of the fibrils. These results coincide with previous results using transmission electron and atomic force microscopy, suggesting an internal fibral-like structure in spider silk [21, 24, 38].

**Figure 2:**
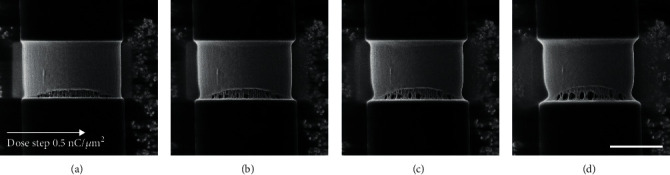
HIM images of a MiS fiber with continuous milling performed with a He^+^ dose of 0.5nC/*μ*m^2^ between each successive image. A tear beginning at the bottom of the cut develops upon increasing milling. Fibral structures are seen to become stretched, while faults/cavities in the sample are seen to form and grow. The fiber is seen to elongate as the milled region stretches. Necking is also observed in the fiber where the tear is forming.

**Figure 3:**
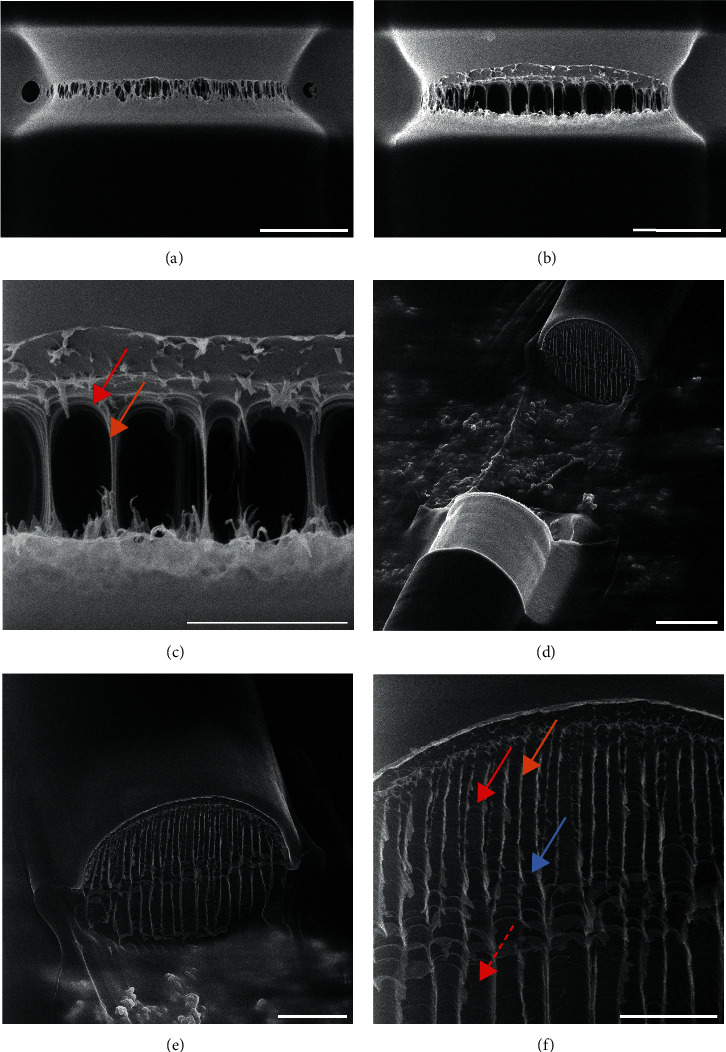
HIM of a MiS fiber cut in half by He^+^ ion sputtering. (a) HIM image of a MiS fiber before breaking. (b) An image of the fiber stretching. (c) Further zoom in on (b), showing the individual fibrils being stretched (orange arrow), causing deformation in the base of the fibril (red arrow). (d) The fiber after breaking—image at 48 degrees. (e) Zoom in on the inner surface of the broken fiber at a 38 degree angle. (f) Further zoom in on the surface (38 degree angle) of the broken fiber, showing the stretched fibril material (orange arrow), the deformed fibril base (red arrow) that becomes wider at the bottom of the fiber (dashed red arrow), and the stacking of the fibrils (blue arrow).

The same experiment was conducted on a MiS fiber where continued milling led to a tear appearing in the silk at one side of the cut (see Figure 2). The tear continued to develop over time and with continued milling. Clear faults/cavities in the fiber are seen to develop, as well as an elongation along the fiber's long axis and a narrowing of the fiber at the forming fault, a process also known as necking [39]. The depicted results are due to a combination of the mechanical and structural properties of spider silk along with the impact of He^+^ ions on the integrity of the fibers. The effect of the He^+^ ion milling reduces the structural integrity of the silk. This results in tension in the spider silk, causing the silk to stretch and even tear at the fault made by the He^+^ ion milling, as seen in Figure 3. This allows visualization of the rupturing process in the fiber.

While the fiber was extending, it was observed that the fibrils were stretched due to the stress (Figures 3(a)–3(c), orange arrow). While the fibrils were stretched forward along with the fiber, they were still bound to the neighbouring fibrils, causing deformation/stretching at the base of the fibrils. This is seen as the bright rounded stripes at the base of the extended structure in Figure 3(c) (red arrow). In Figure 3(c), the inherent depth of field of the HIM is visible, showing multiple fibrils at different depths.

After continued milling, the fibers tear and separate into two pieces. One end of the fiber retracts several *μ*m, making it possible to image the fiber's cross-section and reveal the inner structure (Figures 3(d)–3(f)). The tearing of the fiber is believed to be due to the inherent tension of the spider silk or the silk fiber being slightly taut when placed upon the carbon tape.

Interestingly, the ends of the fiber are relatively even and are torn at a right angle to the long access of the fiber. Figures 3(e) and 3(f) show a characteristic layered structure of the fibers consisting of an outer ring and vertical stripes with smaller and dimmer connecting bands. The outer ring has a diameter of several hundred nm (300-600 nm), which could be due to the proposed layered structure of the silk, where an outer skin layer with a different protein composition is found [38].

When examining the structure within the silk fiber, bright vertical lines (Figure 3(f), orange arrow) and dark ridges (Figure 3(f), red arrow) could be seen perpendicular to the fiber length, which becomes wider towards the bottom of the fiber (Figure 3(e), dashed red arrow).

It is proposed that the brightly striped structures seen on the ends of the fibers are formed by the regions of connection between the two fiber ends before rupturing, also seen in Figure 3(c) (orange arrow).

Therefore, the bright vertical lines seen in Figures 3(d)–3(f) originate from where the silk was stretched and elongated and comprise the remaining material from the individual fibrils that were stretched and ruptured. The dark vertical areas are formed by the vacancies, as also seen in Figure 3(c) (red arrow).

Examination of Figure 3(f) reveals rounded horizontal structures with a typical width of 100-200 nm, corresponding to the expected diameter of the fibrils (blue arrow). The structures are believed to be the outlines of the base of individual fibrils (seen as the dimmer horizontal lines, Figure 3(f), blue arrow). They are, however, stretched and seen to migrate into the remains of the connective regions, suggesting a redistribution of material to these regions while the fiber is stretching.

Remarkably, the connective structures and cavities propagate vertically down throughout the whole fiber, are aligned vertically, and mutually parallel to each other. Furthermore, the outline of the base of each fibril also suggests that the fibrils are stacked one upon another, while it was seen before that the fibrils are randomly or hexagonally stacked (Figure 3(a)).

An explanation could be that faults form at the top of the fiber with typical size and spacing, which seems to correspond to the size of the fibrils, suggesting the rupture of specific fibrils could be forming the initial faults. Therefore, it is proposed that upon cutting the fiber, the weakest points of the fiber create defects/vacancies at the top of the fiber, which propagate throughout the fiber, causing a rearrangement of the fibrils. This rearrangement, in turn, causes the fibrils to appear ordered. The orientation of the connective regions originates from the cutting and rupturing of the fiber from above.

As the He^+^ ions milled the fiber from the top, the top part was broken and/or cut first, and less stress was exerted on these fibrils. The fibrils on the bottom were broken last, and the stress and elongation on these were higher, causing a larger deformation of the base of the fibrils and making the ridges broader. Furthermore, the increased stress on the bottom half of the fiber can be seen in the deformation of the bottom part of the fiber seen in Figure 3(e). It cannot be excluded that the effects seen could also be due to residual heat degradation of the silk. However, we have used a very low current of 1 pA for milling and found that changing the milling beam overlap from 50% to 20% gave no apparent differences. This leads us to believe that the residual heat damage, if present, was minimal.

## 4. Conclusion

The first decade of bioimaging using HIM was mainly focused on comparing ion imaging and electron microscopy. One can list several advantages of He^+^ ion-based imaging, going beyond the capabilities available through a traditional SEM. In addition, both He^+^ and Ne^+^ ions could be utilized for milling and cross-sectioning at subnanometer resolution. The main advantage of HIM milling over conventional focused gallium ion milling of biological samples is simple point-and-shoot milling, which allows for soft milling with reduced heat destruction of the materials.

This work showed that HIM is a valuable tool for visualizing biological samples. The sputtering of the He^+^ ions could be used to softly mill the samples without too much damage to the biological structures. This was in contrast to the Ne^+^ ions, which caused damage to the spider silks' internal structure.

It was also shown that combining He^+^ ion milling and the tension in the spider silk sample made it possible to cut the specimen and visualize the rupturing process and the internal structures in the silk. This method could be used for other fibrous structures or even with nonelastic samples if used with stretched adhesive carbon tape, which could be used to apply the rupturing force.

The HIM images of the spider silk revealed that the rupturing process was highly dynamic, involving rearrangement of the material in the fiber and showing strong indications of an internal fibril structure in the silk fibers with typical dimensions of 100-200 nm.

In the future, we anticipate that HIM will significantly contribute to some of the most challenging imaging applications and, when paired with other imaging modalities, may open new directions in future bioimaging.

## Data Availability

The images used to support the findings of this study are included within the article. Supporting images can be provided upon request to the authors.

## Supplementary Materials

Supplementary Materials Supplementary Figure S1: showing the effect of milling with Ne^+^ ions. (a) a MAS fiber before milling. (b) The same fiber after milling. No internal structures (fibrils) are seen. (c) MiS fiber after milling. Damage to the sample is clearly seen, and no internal structure is visible. Scale bars are 1 *μ*m.
