# Supplementary material for: Helium Ion Microscopy and Sectioning of Spider Silk
Source: Scanning. 2023 May 22;2023:2936788. doi: 10.1155/2023/2936788 (PMC10228223; doi:10.1155/2023/2936788)
Supplement: Supplementary Materials — Supplementary Figure S1: showing the effect of milling with Ne+ ions. (a) a MAS fiber before milling. (b) The same fiber after milling. No internal structures (fibrils) are seen. (c) MiS fiber after milling. Damage to the sample is clearly seen, and no internal structure is visible. Scale bars are 1 μm. [file 2936788.f1.docx]

**Supplementary materials for**

Helium ion microscopy and sectioning of spider silk

Irina Iachina^1,2^, Jonathan R. Brewer^2*^, Horst-Günter Rubahn^1^, Jacek Fiutowski^1^

^1^NanoSYD, Mads Clausen Institute, University of Southern Denmark

^2^Department of Biochemisty and Molecular Biology, University of Southern Denmark

* Corresponding author. Email: brewer@bmb.sdu.dk

**
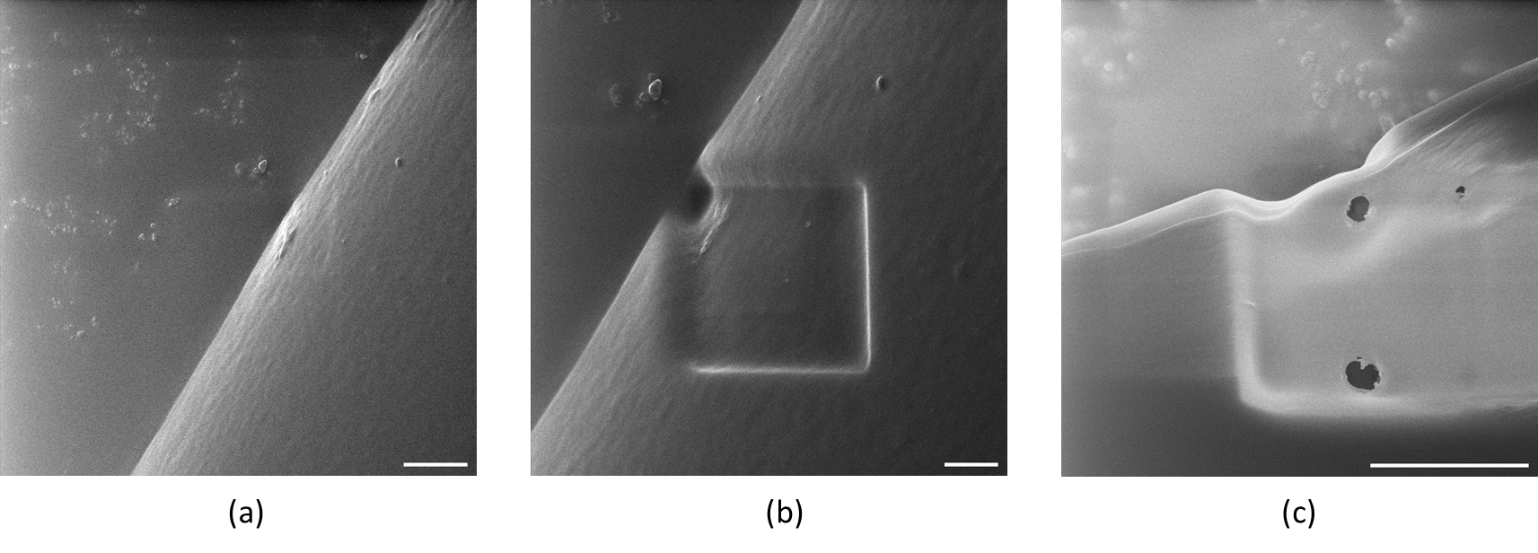
**

***Supplementary figure S1:*** *Showing the effect of milling with Ne^+^ ions. a) a MAS fiber before milling. b) the same fiber after milling. No internal structures (fibrils) are seen. c) MiS fiber after milling. Damage to the sample is clearly seen, and no internal structure is visible. Scale bars are 1µm.*
